# Optimal doses of caspofungin during continuous venovenous hemodiafiltration in critically ill patients

**DOI:** 10.1186/s13054-016-1594-9

**Published:** 2017-01-30

**Authors:** Gerardo Aguilar, Rafael Ferriols, Angels Lozano, Carlos Ezquer, José A. Carbonell, Ana Jurado, Juan Carrizo, Ferran Serralta, Jaume Puig, David Navarro, Manuel Alos, F. Javier Belda

**Affiliations:** 1 Surgical Intensive Care Unit, Department of Anesthesiology and Intensive Care, Hospital Clínico Universitario de Valencia, Avenida Blasco Ibáñez, 17, 46010 Valencia, Spain; 2 Department of Pharmacy, Hospital Clínico Universitario de Valencia, Avenida Blasco Ibáñez, 17, 46010 Valencia, Spain; 3 Instituto de Investigación Sanitaria, INCLIVA, Avenida Blasco Ibáñez, 17, 46010 Valencia, Spain; 4 Department of Microbiology, Hospital Clínico Universitario de Valencia, Avenida Blasco Ibáñez, 17, 46010 Valencia, Spain; 5 School of Medicine, University of Valencia, Avenida Blasco Ibáñez, 15, 46010 Valencia, Spain

**Keywords:** Echinocandins, Continuous renal replacement therapy, Invasive candidiasis

The aim of the present study was to describe the pharmacokinetics of caspofungin in 12 critically ill adult patients with suspected or proven invasive candidiasis who were receiving continuous venovenous hemodiafiltration (CVVHD).

CVVHD was performed using a polysulfone hemofilter (Fresenius, Germany). Caspofungin was administered at usual doses. Pre-filter and post-filter blood, ultradiafiltrate, and urine samples were collected at steady state on day 3 or later, before the dose infusion started, and 0.5, 1, 1.5, 2, 4, 6, 8, and 24 h after the infusion ended.

The drug concentrations were measured by high performance liquid chromatograpy (HPLC) and the following pharmacokinetic parameters were calculated: area under the concentration-time curve (AUC_0-24h_), elimination t_1/2_, volume of distribution (Vd), clearance, trough concentration (C_trough_), and maximum concentration (C_max_).

The results of our study are summarized in Tables 1 and 2 and Fig. 1. Caspofungin was negligible in the ultradiafiltrate and urine samples, confirming the lack of drug elimination through hemofiltration or hemodialysis. Similar findings were previously described by Weiler et al. [1]. Additionally, the mean concentration of caspofungin was slightly higher in the post-filter line than in the pre-filter line (Fig. 1), allowing us to rule out the adsorption to the filter hypothesized in other studies with echinocandins [2, 3].

**Table 1:** Individual arterial caspofungin concentrations (mg/L) of the 12 patients studied

Time (h)	1	2	3	4	5	6	7	8	9	10	11	12
Predose	3.09	2.12	2.94	0.90	1.50	3.04	2.10	2.93	2.18	3.16	2.69	2.62
0.5	10.85	6.96	8.50	4.38	4.59	9.86	7.09	8.23	7.81	11.17	10.24	7.02
1	9.34	6.19	8.23	2.80	4.44	9.11	6.10	7.23	6.69	9.91	8.88	5.78
1.5	8.55	5.75	7.05	NA	4.41	8.24	5.27	6.04	6.03	8.42	8.39	5.09
2	7.51	5.47	6.91	2.43	3.85	7.37	4.96	5.88	5.72	7.74	7.92	4.61
4	6.38	4.49	6.13	2.12	3.77	6.54	4.13	5.66	5.32	6.94	6.62	3.94
6	5.63	3.96	5.63	NA	3.04	5.84	3.54	5.33	4.55	6.40	6.31	3.60
8	5.00	3.40	5.22	1.99	2.80	4.71	3.10	4.45	4.49	5.61	6.00	3.27
24	3.47	2.30	3.10	1.34	1.59	2.47	1.63	2.73	2.27	2.88	4.00	1.85

Time refers to the time since caspofungin infusion ended. *NA* data not available

**Table 2:** Pharmacokinetics of caspofungin during continuous venovenous hemodiafiltration in 12 patients

	AUC_0-24_ (mg h/L)							
Patient	Arterial	Venous	Difference venous to arterial (%)	Vd (L)	Cl (L/h)	C_max_ (mg/L)	C_trough_ (mg/L)	t_½_ (h)
1	140.0	180.0	29	14.1	0.356	12.5	3.47	27.4
2	88.3	106.0	20	17.1	0.567	7.8	2.1	21.0
3	124.0	152.0	23	10.9	0.402	8.8	3.1	18.8
4	65.4	77.4	18	26.8	0.765	6.9	1.3	24.3
5	68.0	90.0	32	17.5	0.735	4.8	1.5	16.5
6	102.0	107.0	5	13.6	0.683	10.7	2.5	13.8
7	65.6	78.8	20	15.0	0.762	8.3	1.6	13.6
8	100.0	113.0	13	13.9	0.499	9.5	2.7	19.3
9	102.0	127.0	25	14.1	0.685	9.2	2.3	14.3
10	121.0	142.0	17	12.5	0.578	12.6	2.9	15.0
11	190.0	224.0	18	13.9	0.368	11.5	4.0	26.2
12	60.1	74.5	24	27.7	1.165	8.5	1.9	16.5
Mean ± SD	102 ± 46	123 ± 46	20.3 ± 7.2	16.4 ± 5.4	0.630 ± 0.225	9.3 ± 2.3	2.4 ± 0.8	18.9 ± 4.9

*SD* standard deviation

**Fig. 1:**
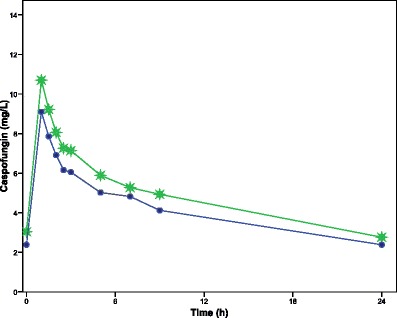
Average caspofungin concentration over time. Infusion started at 0 h and continued over 1 h. *n* = 12 patients. *Solid dots*, arterial; *asterisks*, venous. (The figure is original for this article)

In four patients (33%), the trough concentration of caspofungin was lower than the MIC_90_s published for *Candida* and *Aspergillus* spp., including *Candida parapsilosis* (2 mg/L) [4]. On the other hand, among echinocandins, micafungin has been associated with 1 log kill/24 h in a murine model of disseminated candidiasis when an AUC/MIC of 865, 450, or 1185 is achieved for *Candida albicans*, *Candida glabrata*, or *C. parapsilosis*, respectively [5]. Taking into account a MIC of 0.1 mg/L [4], and using the target pharmacokinetics/pharmacodynamics (PK/PD) described for micafungin, we would have reached this concentration in only nine patients (75%, AUC > 86.5 mg h/L) for *C. albicans* and four patients (33%, AUC > 118.5 mg h/L) for *C. parapsilosis* but all patients for *C. glabrata* (AUC > 45 mg h/L) (Table 2). These data suggest that caspofungin dosing could be insufficient in some critically ill patients.

In conclusion, CVVHD appears to have a negligible effect on caspofungin clearance. However, the licensed regimen of caspofungin was not adequate to reach the PK/PD targets in some critically ill patients, regardless of the use of CVVHD. Nevertheless, future studies are needed to confirm these findings.

## Abbreviations

AUC: Area under the concentration-time curve
C_max_: Maximum concentration
C_trough_: Trough concentration
CVVHD: Continuous venovenous hemodiafiltration
MIC_90_: Minimum inhibitory concentration required to inhibit the growth of 90% of a microorganism
PK/PD: pharmacokinetic/pharmacodynamic
Vd: Volume of distribution
